# *Lef1* Haploinsufficient Mice Display a Low Turnover and Low Bone Mass Phenotype in a Gender- and Age-Specific Manner

**DOI:** 10.1371/journal.pone.0005438

**Published:** 2009-05-04

**Authors:** Tommy Noh, Yankel Gabet, Jon Cogan, Yunfan Shi, Archana Tank, Tomoyo Sasaki, Braden Criswell, Alexis Dixon, Christopher Lee, Joseph Tam, Thomas Kohler, Eran Segev, Lisa Kockeritz, James Woodgett, Ralph Müller, Yang Chai, Elisheva Smith, Itai Bab, Baruch Frenkel

**Affiliations:** 1 Department of Biochemistry and Molecular Biology, Keck School of Medicine, University of Southern California, Los Angeles, California, United States of America; 2 Institute for Genetic Medicine, Keck School of Medicine, University of Southern California, Los Angeles, California, United States of America; 3 Center for Craniofacial Molecular Biology, Keck School of Medicine, University of Southern California, Los Angeles, California, United States of America; 4 Bone Laboratory, Faculty of Dental Medicine, The Hebrew University of Jerusalem, Jerusalem, Israel; 5 Institute for Biomechanics, ETH Zürich, Zürich, Switzerland; 6 Ontario Cancer Institute/Princess Margaret Hospital, Toronto, Ontario, Canada; 7 Department of Orthopaedic Surgery, Keck School of Medicine, University of Southern California, Los Angeles, California, United States of America; University of Michigan, United States of America

## Abstract

We investigated the role of *Lef1*, one of the four transcription factors that transmit Wnt signaling to the genome, in the regulation of bone mass. Microcomputed tomographic analysis of 13- and 17-week-old mice revealed significantly reduced trabecular bone mass in *Lef1^+/−^* females compared to littermate wild-type females. This was attributable to decreased osteoblast activity and bone formation as indicated by histomorphometric analysis of bone remodeling. In contrast to females, bone mass was unaffected by *Lef1* haploinsufficiency in males. Similarly, females were substantially more responsive than males to haploinsufficiency in *Gsk3β*, a negative regulator of the Wnt pathway, displaying in this case a high bone mass phenotype. *Lef1* haploinsufficiency also led to low bone mass in males lacking functional androgen receptor (AR) (*tfm* mutants). The protective skeletal effect of AR against Wnt-related low bone mass is not necessarily a result of direct interaction between the AR and Wnt signaling pathways, because *Lef1^+/−^* female mice had normal bone mass at the age of 34 weeks. Thus, our results indicate an age- and gender-dependent role for *Lef1* in regulating bone formation and bone mass *in vivo*. The resistance to *Lef1* haploinsufficiency in males with active AR and in old females could be due to the reduced bone turnover in these mice.

## Introduction

The wingless/Wnt family of secreted glycoproteins has critical roles in cell growth and differentiation, and is highly conserved among vertebrates, flies, and primitive multicellular organisms [1], [2]. In mammals, the canonical Wnt pathway is pivotal to embryogenesis and tumorigenesis as well as in the maintenance and regeneration of tissues such as skin, intestine, liver, cardiac muscle, and the nervous system [1]–[7]. Wnt ligands bind to membrane complexes consisting of a seven transmembrane domain receptor of the frizzled family [3]–[5] and a ‘single-pass’ co-receptor, Lipoprotein Receptor-Related Protein 6 (Lrp6) and possibly Lrp5 as well [6]–[8]. In the absence of stimulation, the Wnt pathway is under the negative control of a pair of protein-serine kinases, Glycogen Synthase Kinase 3α and 3β (Gsk3α, Gsk3β), which phosphorylate β-catenin, a Wnt transducer, resulting in its ubiquitination and subsequent proteasomal degradation [9], [10]. Upon Wnt stimulation, the Gsk3-mediated phosphorylation of β-catenin is attenuated through disruption of the β-catenin destruction complex [11]. Subsequently, β-catenin accumulates and translocates to the nucleus, where it activates Wnt target genes by associating with the DNA-binding HMG box transcription factors lymphoid enhancer factor 1 (Lef1), T-cell factor 7 (Tcf7), Tcf7L1, and/or Tcf7L2 [12]–[15]. Tcf7, Tcf7L1 and Tcf7L2 are commonly known as Tcf1, Tcf3, and Tcf4, respectively, and these common names are used in this paper.

Osteoporosis, the most prevalent degenerative disease in western societies, is characterized by decreased bone mass and structural integrity. The mammalian skeleton undergoes continuous turnover, where overall bone gain or loss is determined by the difference between bone resorption and formation. After birth, bone mass increases until it reaches “peak bone mass”, which is then maintained at a constant level during young adulthood, followed by an age-related bone loss [16]. Bone mass accrual is subject to sexual dimorphism, with males having higher trabecular bone volume density and lower bone turnover than females [16]–[19]. The age-related bone loss is associated with reduction in turnover rate especially in females [20], [21].

In humans and mice alike, bone mass is strongly dependent on the Wnt signaling pathway [22], [23]. Mutations and polymorphism in *Wnt10b*, *Dkk1*, *Dkk2*, *Sfrp1*, *Sost*, *Lrp6*, and *Gsk3β* affect osteoblast function and therefore bone formation and bone mass [24]–[30]. The Wnt pathway has also been implicated in mediating positive and negative control of bone formation and bone mass by environmental factors such as pharmacological glucocorticoids [31]–[34] and mechanical stimulation [35]. Interestingly, Wnt signaling in osteoblasts also controls osteoclast activity, as demonstrated by increased resorption and a low bone mass (LBM) phenotype in mice whose osteoblasts lack β-catenin or Tcf1 [23]. The role of Lef1 in postnatal bone metabolism has not been studied, in part because *Lef1* deficient mice die within the first week of life [36].

During early embryogenesis, Tcf1 and Lef1 are redundant. Mice lacking both genes have multiple defects, including duplicated neural tubes and malformed limb buds, whereas mice lacking either gene alone do not exhibit these defects [37], [38]. Non-redundant functions of these transcription factors are suggested by the distinct phenotypes observed in mice lacking either Tcf1 or Lef1. Whereas *Tcf1*-null mice display attenuated T cell differentiation [39], *Lef1*-null mice exhibit developmental defects in teeth, hair follicles, mammary glands and the brain [36], [40]. In terms of skeletal metabolism, analysis of *Tcf1*-null mice disclosed accelerated bone resorption [23], but similar analysis of *Lef1*-null mice is hampered by their perinatal lethality. In this study, we employed the viable *Lef1* haploinsufficient mice, which do not display the abnormalities observed in the null mice, to assess the role of *Lef1* in postnatal bone metabolism. We demonstrate a low bone mass phenotype in *Lef1^+/−^* mice, which appears to manifest specifically in the context of high bone turnover rate.

## Results

### Low bone mass (LBM) phenotype in *Lef1* heterozygous female mice

**Figure 1:**
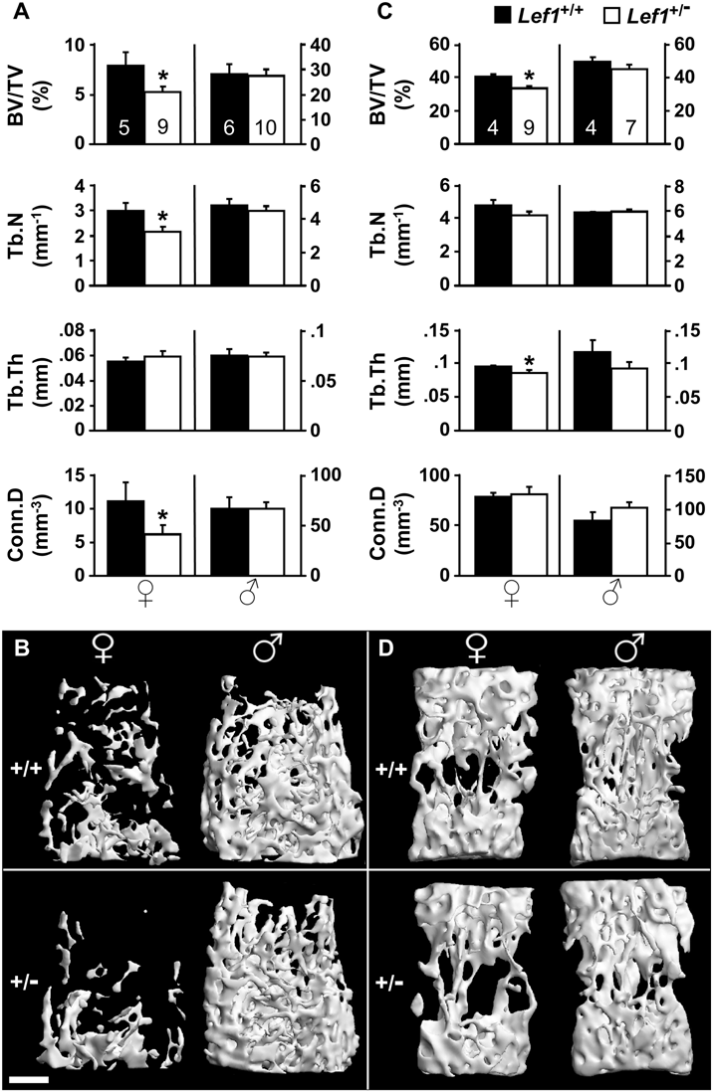
Low trabecular bone mass in *Lef1^+/−^* female mice. (A,C) μCT analysis of the distal femoral (A) and the vertebral (C) trabecular bone of *Lef1^+/+^ (black)* and *Lef1^+/−^ (white)* female (*left*) and male (*right*) 13-week old mice. BV/TV – trabecular bone volume density, Tb.N – trabecular number, Tb.Th – trabecular thickness, Conn.D – connectivity density (Mean±SEM of 4–10 specimens as indicated within the bars at the top, * = *p*<0.05). (B,D) μCT images of distal femoral (B) and vertebral (D) trabecular bone of female (*left*) and male (*right*) 13-week old mice with median BV/TV. Scale bar = 0.5 mm.

Micro-computed tomographic (μCT) analysis of 13-week old *Lef1* haploinsufficient female mice revealed a LBM phenotype compared to littermate controls (Figure 1). The trabecular bone volume density (BV/TV) measured in the distal femur (Figure 1A) and the vertebral body (Figure 1C) of *Lef1* haploinsufficient mice was 34% and 17% lower than the wild-type (WT) controls, respectively. In contrast to females, male mice showed no difference between *Lef1^+/−^* and WT animals (Figure 1). The decrease in BV/TV due to *Lef1* haploinsufficiency resembles the magnitude of trabecular bone loss due to *Lrp6* haploinsufficiency [24].

Detailed analysis of the trabecular bone parameters in the 13-week old female mice revealed interesting site-specific responses to *Lef1* haploinsufficiency. While the decreased BV/TV at the vertebral bodies was attributable to thinning of trabeculae, the LBM at the distal femoral metaphysis was due to decreased trabecular number (Tb.N) and was also associated with decreased connectivity density (Conn.D, Figure 1). Skeletal site-specific control of bone mass, both related and unrelated to the Wnt signaling pathway, has been previously observed [25], [41]–[44], although the underlying mechanisms remain to be elucidated. A similar trabecular bone phenotype was observed in 17-week old *Lef1^+/−^* females, and again males were unaffected. At 13 weeks of age, the *Lef1^+/−^* females, but not males, also exhibited reduced cortical bone thickness (0.162 mm *vs.* 0.188 mm in wild-type females, p = 0.021), but the femoral length and mid-diaphyseal diameter were unaffected by the *Lef1* gene dosage in either gender (data not shown). Additionally, we did not detect any skeletal abnormalities in *Lef1* knockout newborns, as indicated by whole mount staining and histological analysis (Figure S1). The total body weight was similar in *Lef1* heterozygous and gender/age-matched WT controls (data not shown). Thus, *Lef1* haploinsufficiency leads to a LBM phenotype specifically in females, demonstrating for the first time a role for *Lef1* in bone metabolism.

### Decreased bone formation in *Lef1^+/−^* female mice

**Figure 2:**
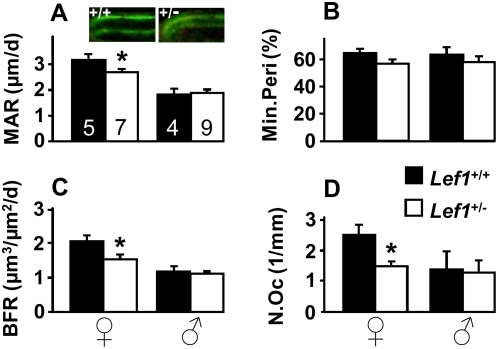
Low bone formation in *Lef1^+/−^* female mice. Histomorphometric analysis of the distal femoral metaphysis from *Lef1* ^+/+^ (*black*) and *Lef1^+/−^* (*white*) female (*left*) and male (*right*) 17-week old mice. (A) mineral apposition rate (MAR), a surrogate for osteoblast activity; images show representative histological sections from *Lef1* ^+/+^ (*left*) and *Lef1^+/−^* (*right*) females; (B) mineralizing perimeter (Min.Peri), a surrogate for osteoblast number; (C) bone formation rate (BFR); (D) trabecular bone osteoclast number (N.Oc/BS). Data represent mean±SEM of 4–9 specimens as indicated within the bars in A; * = *p*<0.05.

Wnt signaling has been implicated in both promoting osteoblast [45] and attenuating osteoclast function [23]. Accordingly, *Lef1* haploinsufficiency could lead to a LBM phenotype by either inhibiting bone formation or stimulating bone resorption. We therefore assessed trabecular bone formation and resorption in distal femoral metaphyses of 17-week old *Lef1^+/−^* mice and littermate controls using vital calcein labeling and TRAP staining, respectively. As shown in Figure 2C, *Lef1^+/−^* female mice exhibited a 25% lower bone formation rate (BFR) as compared to WT controls (Figure 2C), attributable mainly to decreased mineral apposition rate (MAR; Figure 2A), which represents the activity of the average osteoblast. Thus, the female *Lef1^+/−^* LBM phenotype is attributable to reduced osteoblast function. In contrast, there was no indication for increased bone resorption in *Lef1^+/−^* females because they had less, not more TRAP-positive cells compared to controls (Figure 2D). Importantly, the skeletal remodeling analysis in male mice revealed no difference between *Lef1^+/−^* and *Lef1* ^+/+^ animals (Figure 2). On a side note, our findings demonstrate lower bone turnover in WT male compared to WT female mice (−42.1% MAR; −51.4% BFR and −45.6% osteoclast number, *p*<0.05 for each parameter), which is consistent with previous reports [46], [47]. The higher bone turnover in females compared to males may predispose the formers to *Lef1* haploinsufficiency-induced LBM (see below).

**Figure 3:**
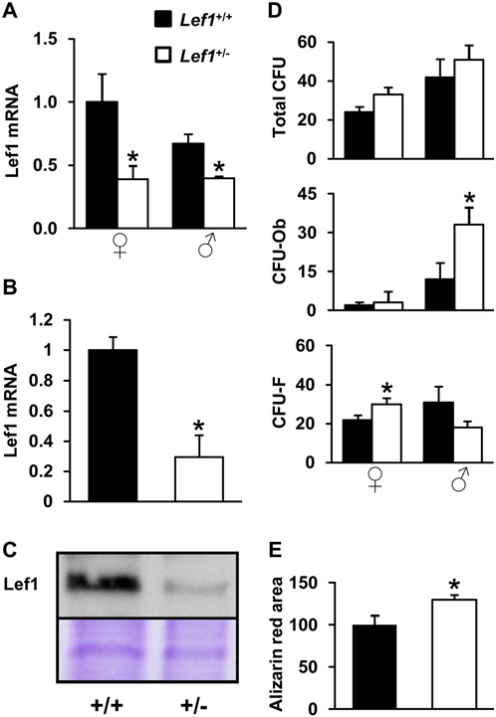
Reduced Lef1 expression is not associated with impaired osteoblast differentiation *in vitro*. (A,B) Lef1 mRNA in tibia of *Lef1* ^+/−^ and control male and female mice (A) and in NeMCO cultures (B) was assessed by RT-qPCR and corrected for the expression of GAPDH and rpL10A, respectively. Bars represent relative expression levels (Mean±SEM, n = 3). (C) Western blot analysis of Lef1 expression in NeMCO cultures. Equal loading was demonstrated by Coomassie blue staining (bottom panel shows a ∼60 KDa Coomassie blue-stained band). (D) Bone marrow mesenchymal cells from WT and *Lef1^+/−^* mice of each gender were cultured for 28 days, fixed and stained with Alizarin red. Colonies were manually counted in each of 6–11 independent cultures for each condition. Alizarin red-positive colonies were counted as osteoblastic colony-forming units (CFU-Ob), and the rest were considered fibroblastic CFU (CFU-F). (E) Day-20 NeMCO cultures from WT and *Lef1^+/−^* mice were fixed and stained with Alizarin red. Data represent mean mineralized area relative to WT±SEM in at least 3 cultures per condition. * = *p*<0.05 *vs.* WT.

We next assessed the effects of *Lef1* heterozygocity on Lef1 expression and on osteoblast differentiation *in vitro*. First, we confirmed that Lef1 expression was significantly reduced in bones and in newborn mouse calvarial osteoblast (NeMCO) cultures derived from *Lef1* heterozygous compared to control mice (Figure 3A–3C). In mesenchymal stem cell (MSC) cultures derived from bone marrow of female mice, *Lef1* haploinsufficiency increased the number of CFU-F, whereas the number of CFU-Ob was unchanged (Figure 3D). Interestingly, *Lef1* haploinsufficiency in male mice, which did not reduce bone mass *in vivo* (Figure 1), was associated with an increase in bone marrow-derived CFU-Ob (Figure 3D). *In vitro* osteoblast differentiation as defined by mineralization in NeMCO cultures was accelerated by *Lef1* haploinsufficiency (Figure 3E).

### High bone mass (HBM) in *Gsk3β* haploinsufficient female mice

**Figure 4:**
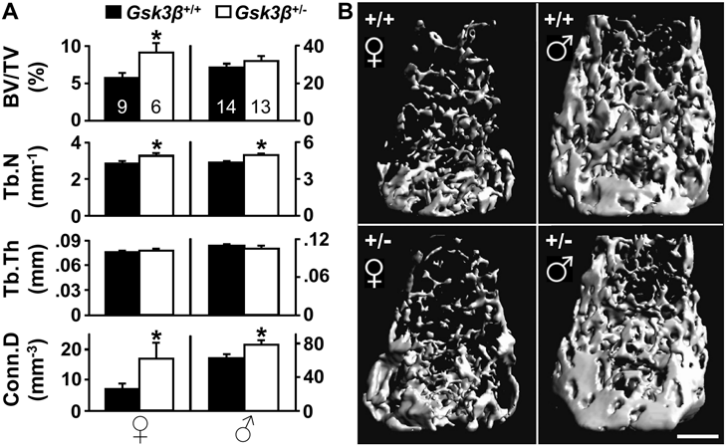
High trabecular bone mass in *Gsk3β* ^+/−^ female mice. (A) μCT analysis of the distal femoral trabecular bone of 16 week old *Gsk3β* ^+/+^ (*black*) and *Gsk3β* ^+/−^ (*white*) female (*left*) and male (*right*) mice. Data represent mean±SEM of 6–14 specimens as indicated within the bars at the top, * = *p*<0.05. (B) μCT images from female (*left*) and male (*right*) mice with median BV/TV. Scale bar = 0.5 mm.

Alterations in components of the Wnt pathway other than Lef1 may also have stronger skeletal effects in females as compared to males. To address this notion, we compared the role of Gsk3β, a negative regulator of the Wnt pathway, in female *versus* male bone mass. Because the *Gsk3ß*-null mice die *in utero* [48], we analyzed the trabecular bone in the distal femoral metaphysis of mice haploinsufficient for *Gsk3ß*. Indeed, female, but not male *Gsk3β* ^+/−^ mice exhibited a high bone mass (HBM) phenotype compared to WT littermates (Figure 4A). The elevated BV/TV was attributable to increased trabecular number, and was associated with increased connectivity density (Figure 4), a mirror image of the respective *Lef1^+/−^* LBM phenotype. Jointly, the gender-preferential effects of both *Lef1* and *Gsk3β* haploinsufficiency suggest that the skeleton is more sensitive to variations in Wnt signaling in females compared to males.

### Androgen signaling protects against *Lef1* haploinsufficiency

**Figure 5:**
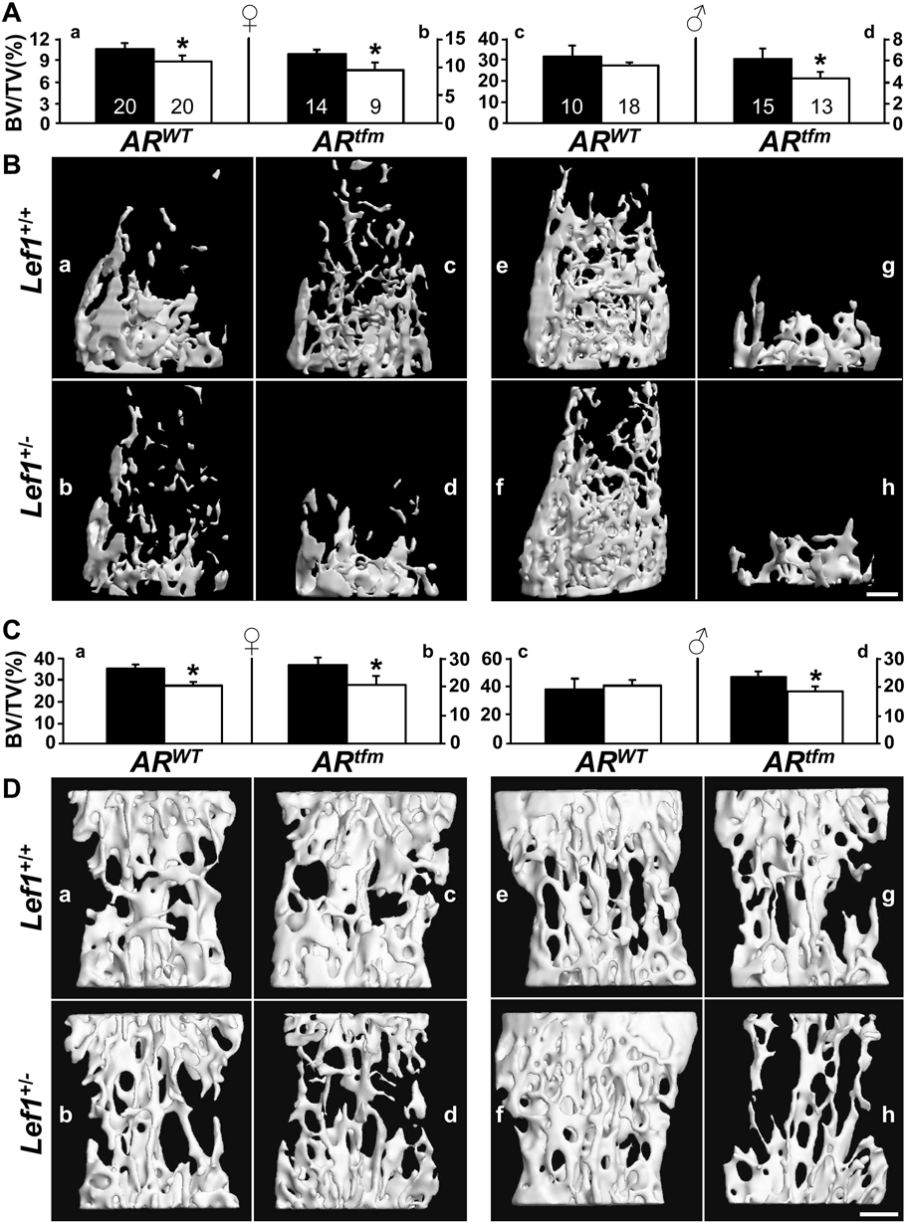
Androgen signaling protects against *Lef1* haploinsufficiency-induced low bone mass. μCT analysis of the trabecular bone compartment in distal femora (A,B) and vertebral bodies (C,D) of 13-week old female (*left*) and male (*right*) *Lef1* ^+/+^ *(black)* and *Lef1^+/−^ (white)* mice. *AR^tfm^* males have no functional AR, while *AR^tfm^* females are carriers for the defective *AR* allele. Data represent mean±SEM of 9–20 specimens as indicated within the bars at the top, * = *p*<0.05. (B) and (D) show respective μCT images of female (*left*) and male (*right*) mice with median BV/TV. Mice in a, b, e and f carry the wild-type AR (*AR^WT^*); mice in c, d, g and h carry the *AR^tfm^* allele; mice in a, c, e and g are *Lef1* ^+/+^; mice in b, d, f and h are *Lef1* ^+/−^. Scale bar = 0.5 mm.

The female-preferential skeletal phenotype of the *Lef1^+/−^* mice could be explained by a compensatory gene(s) on the Y chromosome, hypersensitization by estrogens, or protection by androgens. In support of the latter possibility, androgens can augment Wnt signaling [49], [50], and even stimulate Lef1 expression in osteoblasts (Figure S2) similar to what has been observed in adipocytes [51]. To test the hypothesis that androgen signaling protects against *Lef1* haploinsufficiency-induced LBM *in vivo*, we employed *tfm* male mice, in which androgen signaling is absent due to a naturally occurring mutation in the androgen receptor (AR) [52]. To generate *Lef1* ^+/−^;*AR^tfm^* mice, *Lef1^+/−^* males were bred with female *tfm* carriers. Because the *AR^tfm^* allele is embedded in a tabby genomic sequence [53], we first analyzed the distal femora in offspring with wild type AR, but which are no longer on a pure C57BL/6 background. Similar to the original results (Figure 1), female *Lef1^+/−^* mice that partially carry the tabby genome exhibited a LBM phenotype (Figure 5Aa, and compare Figure 5Ba to 5Bb), while male mice on the same genetic background were protected (Figure 5Ac, and compare Figure 5Be to 5Bf). Remarkably, however, male *tfm* mice were vulnerable to *Lef1* haploinsufficiency, similar to females (Figure 5). Specifically, *Lef1* ^+/−^;*AR^tfm^* mice had a 22% lower BV/TV as compared to their *Lef1* ^+/+^;*AR^tfm^* counterparts (Figure 5Ad, and compare Figure 5Bg to 5Bh). Similar results were observed in the vertebral bodies (Figure 5C and 5D). Because estrogen levels in *tfm* males do not approach those of females [54], and because these mice still carry an intact Y chromosome, these results demonstrate that androgen signaling protects against *Lef1* haploinsufficiency-induced LBM.

### Aged females are resistant to *Lef1* haploinsufficiency-induced LBM

**Figure 6:**
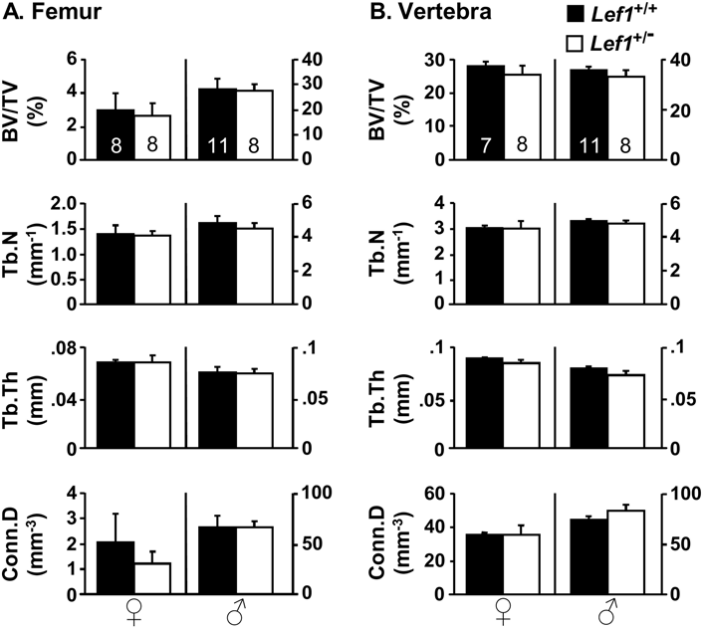
Thirty four-week old female mice are insensitive to *Lef1* haploinsufficiency. (A,B) μCT analysis of the distal femoral (A) and the vertebral (B) trabecular bone of *Lef1^+/+^ (black)* and *Lef1^+/−^ (white)* female (*left*) and male (*right*) 34-week old mice (Data represent mean±SEM of 7–11 specimens as indicated within the bars at the top).

It has been previously suggested that androgen signaling can augment Wnt signaling in bone cells [49], [50]. This could explain how males are protected against *Lef1* haploinsufficiency-induced LBM. Alternatively, androgens could also indirectly protect males by restraining bone turnover [54], [55]. If low bone turnover protects male mice from *Lef1* haploinsufficiency, then *Lef1^+/−^* females may no longer display a low bone mass phenotype compared to WT females at ages older than 17 weeks, when bone turnover decreases [20], [21]. Indeed, μCT analysis of 34-week old female mice revealed no difference between *Lef1^+/−^* and WT females (Figure 6). At this age, the male skeleton was again unaffected by *Lef1* haploinsufficiency (Figure 6). In summary, *Lef1* haploinsufficiency induces LBM in a gender- and age-specific manner.

## Discussion

The present work demonstrates low bone mass in mice, in which one *Lef1* gene copy is disrupted. Interestingly, bone mass was reduced in 13- and 17-week old females, but not in males of any age or in 34-week old females. This gender-specificity reflected neither protection by a Y chromosome-associated gene nor sensitization by estrogens, since *Lef1^+/−^* male mice harboring a testicular feminization mutation (*tfm*) also displayed a LBM phenotype despite their having an intact Y chromosome and not having feminine estrogen levels [54]. *Gsk3β^+/−^* mice displayed a mirror image of the *Lef1^+/−^* phenotype, namely increased bone mass in female mice only. In line with our findings, disruption of *Sfrp1*, a Wnt antagonist, resulted in a female-preferential bone phenotype [29]. Thus, genetic alterations in components of the Wnt pathway appear to affect female more than male mice. That *Lrp5* deficiency reduces bone mass equally in males and females [56] is not inconsistent with our conclusion in light of the recent work of Yadav et *al.* [57], who disputed the paradigm that places Lrp5 upstream of Gsk3β and Lef1 in osteoblasts. Plausibly, a genetic lesion at the level of *Lef1* itself compromises canonical Wnt signaling and is therefore more consequential in females than in males.

All mice that were sensitive to *Lef1* haploinsufficiency in our study, namely young females and *tfm* males, are also characterized by a relatively high rate of bone turnover (Figure 2 and ref. [54]). In contrast, mice resistant to *Lef1* haploinsufficiency—*AR* ^WT^ males and aged females (Figure 1, Figure 5, Figure 6)—have a lower rate of bone turnover (Figure 2 and [20], [21]). Thus, a unifying explanation for our observations is that bone turnover rate determines the skeletal response to genetic alterations in canonical Wnt signaling. Our favored interpretation of the gender-specific sensitivity to *Lef1* haploinsufficiency is that androgens protect the skeleton from the potential deleterious effect of reduced *Lef1* by restraining bone turnover [54], [55].

We cannot rule out an alternative explanation whereby AR activity compensates for *Lef1* haploinsufficiency via molecular interaction with the canonical Wnt pathway. In fact, it has been shown that DHT stimulates Lef/Tcf-mediated transcription in osteoblasts [49], [50]. This could occur via physical interaction of liganded AR with β-catenin [51] or with membrane residents such as Src [58], which could then impinge on the Wnt pathway through activation of the PI3 kinase/Akt/Gsk3β axis [50], [59], [60]. Alternatively, androgens could regulate the expression of either Lef1 itself (Figure S2 and [51]) or Wnt agonists and/or antagonists [61]. However, the normal bone phenotype observed in 34-week old *Lef1^+/−^* female mice favors the hypothesis that androgen signaling, much like aging in females, overrides the skeletal sensitivity to *Lef1* haploinsufficiency by restraining bone turnover. Obviously, the two explanations for AR-mediated protection against *Lef1* haploinsufficiency – molecular interaction with the Wnt pathway and attenuation of bone turnover – are not mutually exclusive.

It remains to be examined to what extent the effect of Lef1 on bone formation is cell autonomous. In favor of cell autonomy is the reduced Lef1 expression in *Lef1* ^+/−^ osteoblasts and the observed gender-dependent changes in the numbers of CFU-F and CFU-Ob in bone marrow-derived MSCs cultures (Figure 3D). Possibly, *Lef1* haploinsufficiency promotes premature osteoblast differentiation ([62], [63] and Figure 3E). However, much like the effect of duodenal Lrp5 [57], the role of Lef1 in regulating bone formation may reside in cells other than osteoblasts. Cell type-specific knockout studies will be necessary to clarify this issue.

An intriguing, albeit speculative, extrapolation from our findings is that females reach lower peak bone mass than males because, in the absence of androgens, higher rate of bone turnover renders the young female skeleton more vulnerable to sub-optimal activity of canonical Wnt signaling and possibly other pathways. Other investigators reported on age- and gender-dependent bone phenotypes in mice with genetic alterations in different pathways. For example, osteoblast-specific disruption of BMP type-IA receptor leads to LBM in young mice but HBM in old mice [64]. Very similar age-dependent effects were reported for *Runx2* haploinsufficiency in mice [20]. In addition, the strong anabolic effect of estrogen in young *Runx2* ^+/−^ mice was almost completely abolished in aged mice [20]. With regard to gender specificity, and in addition to the female-preferential response to genetic manipulation of *Lef1*, *GSK3ß* and *Sfrp1*, ablation of *Cathepsin K* results in a 3-fold stronger effect in female compared to male mice [47]. Age- and gender-related variations in bone turnover may explain the differential skeletal responses to some of these and other genetic aberrations. Furthermore, hormonal and age-related variation in bone turnover may contribute to gender- and age-related susceptibility to osteoporosis and response to therapies.

## Materials and Methods

### Animals

*Lef1^+/−^* and *Gsk3β* ^+/−^ mice and their controls, all on a C57BL/6 background, were generated by breeding *Lef1^+/−^* [36] or *Gsk3β* ^+/−^ [26] mice with C57BL/6 mice from either Harlan Laboratories (Indianapolis, Indiana, USA) or the Ontario Cancer Institute (Toronto, Canada), respectively. Mice carrying the testicular feminization mutation (*Tfm*) (Jackson Laboratories, Bar Harbor, Maine, USA) on a C57BL/6J*-A-Ta<6J>* background were bred with the *Lef1^+/−^* mice and F1 litters were examined. To measure the percentage of bone surface undergoing mineralization and the mineralization rate, mice were injected intraperitoneally with 15 mg/kg of the fluorochrome calcein (Sigma-Aldrich, St. Louis, MO, USA) four days and again one day prior to sacrifice. One femur and the fifth lumbar vertebra (L5) from each mouse were dissected and fixed in 10% phosphate-buffered formalin (pH = 7.2) for 24 hours, and then stored in 70% ethanol. All experiments were approved by the Institutional Animal Care and Use Committee (IACUC) of the University of Southern California and of the University of Toronto.

### Micro-computed tomography

Femora (one per mouse) and fifth lumbar vertebrae (L5) were examined as reported previously [65], [66] using either Scanco μCT 40 (Scanco Medical AG, Brüttisellen, Switzerland), or Siemens MicroCAT II (Siemens Medical Solutions, Knoxville, TN, USA). Briefly, scans were performed at a 20-µm resolution in all three spatial dimensions. The mineralized tissues were differentially segmented by a global thresholding procedure [67]. Trabecular parameters in the secondary spongiosa of the distal femoral metaphysis included trabecular bone volume density (BV/TV), trabecular thickness (Tb.Th), trabecular number (Tb.N) and connectivity density (Conn.D). Cortical thickness, diaphyseal diameter, and medullary cavity diameter were determined in the mid-diaphyseal region. In L5 bodies, the entire trabecular bone compartment was analyzed. All morphometric parameters were determined by using a direct 3D approach [68]. Differences between groups were analyzed by student's *t*-test (two-tailed) and were considered significant when *p*<0.05.

### Histomorphometry

After μCT image acquisition, femora were embedded undecalcified in polymethylmethacrylate (Technovit 9100, Heraeus Kulzer, Germany). Undeplasticized longitudinal 5-µm sections from the center of each bone were left unstained for dynamic histomorphometric measurements. To identify osteoclasts, consecutive sections were deplasticized and stained with tartrate-resistant acid phosphatase (TRAP; Sigma-Aldrich, St. Louis, MO, USA) and counterstained with Mayer's hematoxylin [69]. The morphometric analysis was performed using the Image-Pro Discovery software (Media Cybernetics, Silver Spring, MD, USA). The following parameters were determined: mineral apposition rate (MAR), mineralizing perimeter (Min.Peri), bone formation rate (BFR) and osteoclast number (N.Oc/BS). The terminology and units used for these measurements were according to the convention of standardized nomenclature [70]. Statistical analysis was performed as above.

### Tissue culture

NeMCO cultures were prepared from one day-old pups as described previously [71]. Cells were cultured in 6-well plates for Western blot analysis and in 12-well plates for RT-qPCR and mineralization assays. For the latter, osteogenic medium containing ascorbic acid (50 µg/mL) and β-glycerophosphate (10 mM) was initiated at confluence and alizarin red staining was performed at day 20. For MSC cultures, the cellular content of the bone marrow cavity from two femurs and two tibiae from each mouse was flushed using αMEM and passed through needles with decreasing diameters (down to 25G) to obtain a single cell suspension. Cells were then plated at 3×10^6^ per well in 6-well plates and incubated for 3 days in αMEM (Invitrogen) supplemented with 15% FBS (Gemini Bio-Products, West Sacramento, CA). Starting at day 3, the MSC were cultured in osteogenic medium and stained with Alizarin red after 28 days.

### Lef1 expression

Total RNA was extracted from freshly isolated tibiae of 10 week-old mice. Upon harvesting, one tibia per animal was stabilized in RNA*Later* (Ambion, Austin, TX), homogenized in Trizol (Invitrogen), purified using 1-Bromo-3-Chloropropane and isopropanol, then rinsed in 70% ethanol. RNA from cells was extracted using Aurum Total RNA Mini Kit (Biorad, Hercules, CA). cDNA was produced using Superscript III First Strand cDNA synthesis kit (Invitrogen) and Real-Time PCR was performed using iQ SYBR green supermix (Biorad) and an Opticon 2 real time PCR machine (Biorad). Lef1 mRNA levels in tibiae and NeMCO cultures were corrected for GAPDH and ribosomal protein L10A (rpL10A) mRNA, respectively. Primers used for PCR are listed in Table 1. Western blot analysis of Lef1 in NeMCO cultures was performed essentially as previously described [71] using anti-Lef1 antibody from Cell Signaling (Danvers, MA) and secondary antibodies from Santa Cruz Biotechnology (Santa Cruz, CA).

**Table 1:** Primers for genotyping and RT–qPCR.

**Genotyping**		
Lef1	LPP2.2	5′TGTCTCTCTTTCCGTGCTAGTTC3′
	D8	5′CCGTTTCAGTGGCACGCCCTCTCC3′
	Neo	5′ATGGCGATGCCTGCTTGCCGAATA3′
Sry	Fwd	5′TCATGAGACTGCCAACCACAG3′
	Rev	5′CATGACCACCACCACCACCAA3′
Tfm*	Fwd	5′GTGAAGCAGGTAGCTCTGGG3′
	Rev	5′GTTCTCCAGCTTGATACGGG3′
**RT-qPCR**		
GAPDH	Fwd	5′CCAGAACATCATCCCTGCAT3′
	Rev	5′CTTGCCCACAGCCTTGGCAGC3′
rpL10A	Fwd	5′CGCCGCAAGTTTCTGGAGAC3′
	Rev	5′CTTGCCAGCCTTGTTTAGGC3′
Lef1	Fwd	5′TGAGTGCACGCTAAAGGAGA3′
	Rev	5′ATAATTGTCTCGCGCTGACC3′

* PCR product was digested with *Mwo*I resulting in either a 137 bp (*WT*) or 182 bp (*Tfm*) band.

## Supporting Information

Figure S1 *Lef1* ^−/−^ mice have normal bone development. Histological evaluation of wild type (a–e) versus *Lef1* ^−/−^ (f–j) newborn mice. (a,b,f,g) Alizarin red/Alcian blue staining of craniofacial bones. (c,h) Alizarin red/Alcian blue staining of hind limb and vertebrae. (d,i) H-E staining of longitudinal femoral sections. (e,j) Toluidine blue staining of distal femoral growth plates. Representative images are shown. No abnormality was detected in the *Lef1* ^−/−^ skeletons, except for the previously reported lack of teeth (f) (van Genderen et al. 1994, Genes Dev 8, 2691-703)
(3.47 MB TIF)

Figure S2 DHT stimulates Lef1 expression *in vitro*. MC3T3-E1 osteoblast cultures maintained in phenol-red free αMEM supplemented with 10% charcoal-stripped serum were treated with 30 nM DHT or 100 nM estradiol for 48 hours. Expression of the four members of the Lef/Tcf gene family was assessed by RT-qPCR and corrected for the expression of rpL10A. Bars represent expression levels in the presence of hormone relative to the ethanol vehicle, defined for each gene as 1.
(0.88 MB TIF)
