# Supplementary figures and images for: Lef1 Haploinsufficient Mice Display a Low Turnover and Low Bone Mass Phenotype in a Gender- and Age-Specific Manner
Source: PLoS One. 2009 May 4;4(5):e5438. doi: 10.1371/journal.pone.0005438 (PMC2673053; doi:10.1371/journal.pone.0005438)

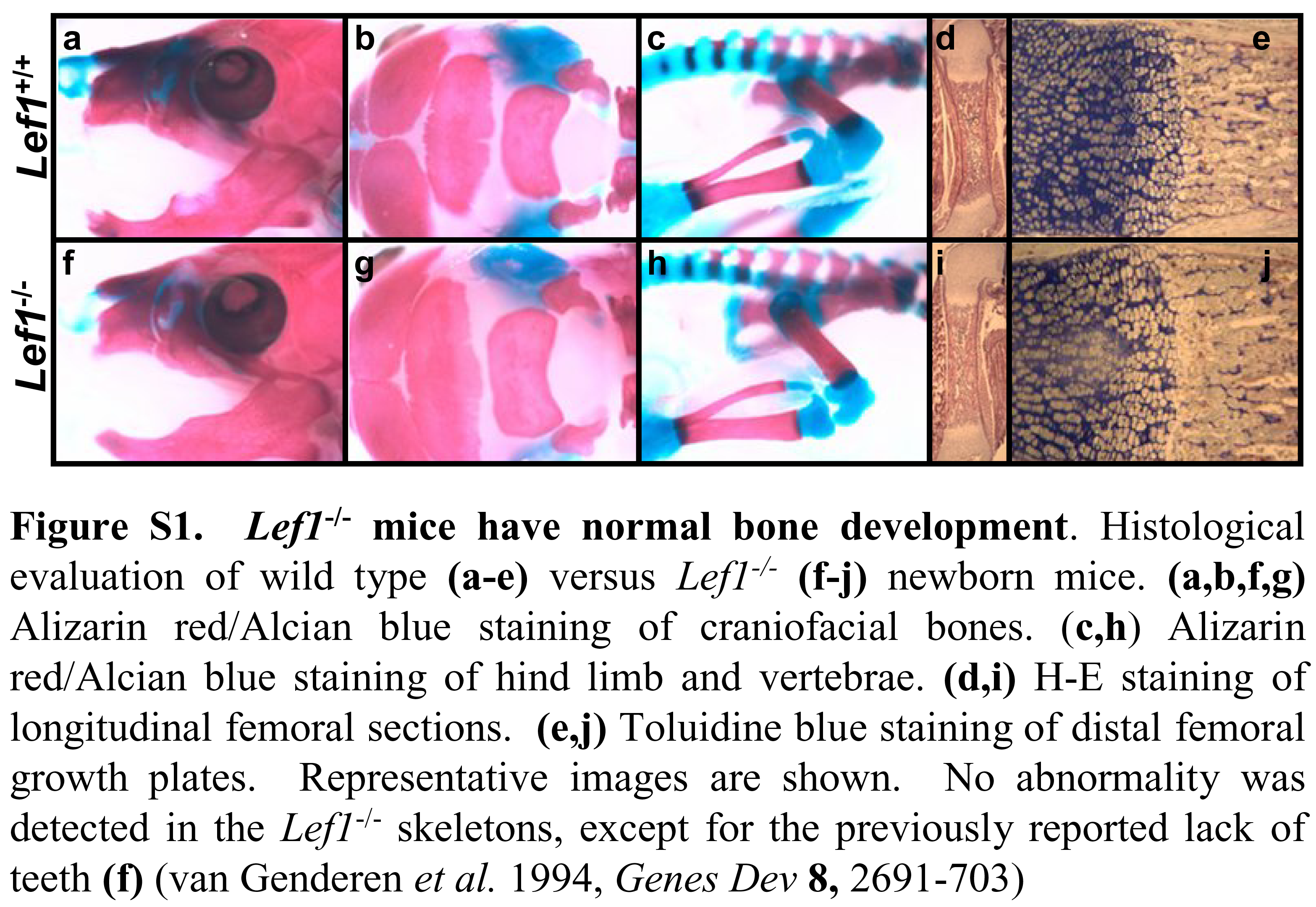

Supplement: Figure S1 — Lef1 −/− mice have normal bone development. Histological evaluation of wild type (a–e) versus Lef1 −/− (f–j) newborn mice. (a,b,f,g) Alizarin red/Alcian blue staining of craniofacial bones. (c,h) Alizarin red/Alcian blue staining of hind limb and vertebrae. (d,i) H-E staining of longitudinal femoral sections. (e,j) Toluidine blue staining of distal femoral growth plates. Representative images are shown. No abnormality was detected in the Lef1 −/− skeletons, except for the previously reported lack of teeth (f) (van Genderen et al. 1994, Genes Dev 8, 2691-703) (3.47 MB TIF) [file pone.0005438.s001.tif]

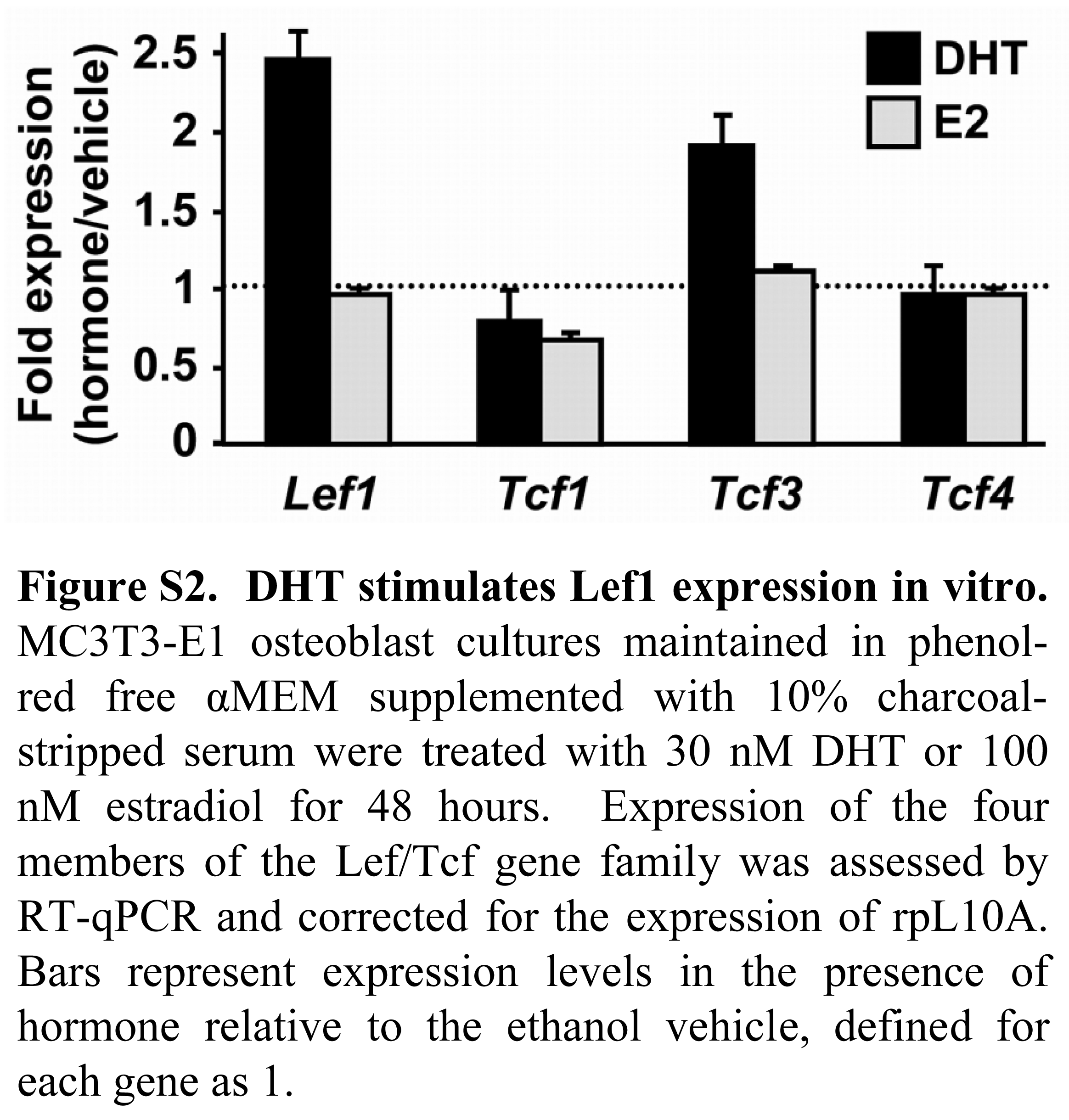

Supplement: Figure S2 — DHT stimulates Lef1 expression in vitro. MC3T3-E1 osteoblast cultures maintained in phenol-red free αMEM supplemented with 10% charcoal-stripped serum were treated with 30 nM DHT or 100 nM estradiol for 48 hours. Expression of the four members of the Lef/Tcf gene family was assessed by RT-qPCR and corrected for the expression of rpL10A. Bars represent expression levels in the presence of hormone relative to the ethanol vehicle, defined for each gene as 1. (0.88 MB TIF) [file pone.0005438.s002.tif]
